# Heparan sulphate expression in high-risk neuroblastoma

**DOI:** 10.1007/s00383-026-06700-3

**Published:** 2026-11-03

**Authors:** Valeria Solari, Lucia Borriello, Jeremy E. Turnbull, Edwin A. Yates

**Affiliations:** 1 Independent researcher, Vienna, Austria; 2 Department of Biochemistry, Cell and Systems Biology, Institute of Integrative Biology, University of Liverpool, Crown Street, Liverpool, L69 7ZB UK; 3 Department of Cancer and Cellular Biology, Lewis Katz School of Medicine, Cancer Signaling and Microenvironment Program, Fox Chase Cancer Center, Philadelphia, USA; 4 Centre for Glycocalyx Research, University of Copenhagen, Copenhagen, Denmark

**Keywords:** Neuroblastoma, Heparan sulphate, SULF2, MYCN amplification

## Abstract

**Purpose:**

Heparan sulphate proteoglycans (HSPGs) regulate tumour cell interactions with the microenvironment. Sulfatase-2 (SULF2), an extracellular endosulfatase that modifies heparan sulphate (HS) 6-O-sulphation, has been implicated in neuroblastoma (NBL), particularly in high risk MYCN-amplified (MYCN-A) tumours. We investigated HS distribution in NBL and the effects of SULF2 knockdown on tumour cell behaviour.

**Methods:**

HS expression was analysed in NBL cell lines using the anti HS antibody 10E4 and the phage display antibody HS3A8V. Cell migration was assessed following siRNA mediated SULF2 knockdown. HS expression in 65 primary human NBL specimens was quantified by colour deconvolution and compared by MYCN status and histological risk groups.

**Results:**

HS epitopes were abundantly expressed on NBL cell surfaces, particularly in MYCN-A SK-N-BE(2) cells and localised along intercellular extensions. SULF2 knockdown significantly reduced cell migration, particularly in MYCN-A cells (*p* < 0.01), altered HS staining and reduced intercellular extensions. In primary MYCN-A tumours showed significantly greater 10E4 immunoreactivity than MYCN-non-amplified (NA) tumours with favourable or unfavourable histology (both *p* < 0.0001), with no difference between the latter groups.

**Conclusion:**

MYCN-A NBL demonstrates a distinct HS phenotype. SULF2-mediated HS remodelling may contribute to tumour cell migration and HS-rich intercellular extensions, identifying SULF2 as a potential therapeutic target in high-risk NBL.

## Introduction

Neuroblastoma (NBL) is the most common extracranial solid tumour of childhood and is characterised by marked biological and clinical heterogeneity, ranging from spontaneous regression or differentiation to rapidly progressive metastatic disease [1, 2]. MYCN amplification (MYCN-A) is associated with aggressive tumour biology and remains an important adverse prognostic factor [3]. Despite advances in multimodal treatment, outcomes for patients with high-risk disease remain unsatisfactory, highlighting the need to better understand the molecular mechanisms regulating tumour cell survival, invasion and interactions with the tumour microenvironment.

The tumour microenvironment is increasingly recognised as an important regulator of NBL progression and therapeutic response. Recent single-cell and spatial profiling studies have demonstrated extensive interactions between NBL cells and immune and stromal populations, highlighting the importance of tumour microenvironment signalling in disease progression and treatment resistance [4–7]. Heparan sulphate proteoglygans (HSPGs), located at the cell surface and within the extracellular matrix, contribute to these interactions by binding a wide range of growth factors, cytokines, chemokines and extracellular matrix proteins [8, 9]. Their biological activity is determined not only by their core proteins but also by the structural diversity of their heparan sulphate (HS) chains. Differential patterns of N-,2-O-, 3-O-, and 6-O-sulphation generate specific binding domains that regulate the localisation, availability and signalling activity of HS-binding proteins, thereby coordinating signalling networks at the cell surface and within the extracellular matrix [8, 10, 11]. Alterations in HS structure may therefore influence cell proliferation, differentiation, adhesion, migration and tumour-stromal interactions.

Evidence suggests that the role of HS in NBL is context dependent. Knelson et al. demonstrated that several HSPGs were expressed at relatively low levels in neuroblasts but enriched within the Schwannian stroma [12]. Soluble HSPGs promoted neuroblast differentiation through FGF2/FGFR1-dependent ERK signalling, while expression of selected HSPGs was associated with favourable clinical outcomes. Furthermore, modified heparin promoted differentiation and reduced tumour growth and metastasis in experimental models [12]. These findings suggested a tumour-suppressive role for stromal HS and highlighted the importance of HS dependent signalling in regulating neuroblast differentiation and malignant progression [12]. The biological effects of HS, however, are also influenced by post-synthetic modification of its sulphation pattern. The extracellular endosulphatases SULF1 and SULF2 selectively remove 6-O-sulphate groups from HS thereby modifying interactions between of HSPGs and signalling molecules [13]. This remodelling can alter growth factor availability and receptor signalling and has been implicated in several malignancies [14]. In NBL, we previously demonstrated a close association between SULF2 and MYCN. SULF2 expression was increased in MYCN-A NBL cell lines and primary tumours, while SULF2 overexpression enhanced the survival of MYCN-non-amplified (MYCN-NA) NBL cells. In independent patient cohorts, increased SULF2 expression was associated with MYCN-A and adverse outcome, suggesting that SULF2 contributes to MYCN-driven tumour biology [15]. Together, these findings suggest that the role of HS in NBL cannot be explained solely by HSPG abundance. Rather, its cellular localisation, structural characteristics and subsequent interactions with proteins may determine whether HS-dependent signalling promotes differentiation or supports malignant behaviour [12, 15]. The association between SULF2 and MYCN further raises the possibility that SULF2-mediated extracellular remodelling generates a distinct HS phenotype in aggressive MYCN-A NBL. However, the distribution of defined HS epitopes in MYCN-A and MYCN-NA NBL cells and primary human tumours, and their relationship to SULF2 activity, remain incompletely characterised. Investigation of HS is complicated by its structural heterogeneity, low immunogenicity and the limited availability of antibodies recognising defined HS structures. Phage-display technology has enabled the generation of single-chain variable fragment antibodies that recognise particular conformational features determined by HS sequence in *situ* [16, 17] and higher-order structural features influenced by cation binding [18]. Together with conventional monoclonal antibodies such as 10E4 [19, 20], these reagents permit complementary characterisation of HS distribution and structural diversity.

In the present study, we used the anti-HS monoclonal antibody 10E4 and the phage-display antibody HS3A8V to investigate HS expression and distribution in MYCN-A and MYCN-NA NBL cells. We further examined the effects of siRNA-mediated SULF2 knockdown on HS distribution, intercellular extensions and NBL cell migration, and analysed 10E4 immunoreactivity in 65 primary human NBL tumours according to MYCN amplification status and histological classification. We hypothesised that MYCN-A NBL is characterised by a distinct HS phenotype associated with SULF2 activity and aggressive tumour cell behaviour.

## Materials and methods

### Cell culture and transfection

Human NBL cell lines SK-N-BE(2) (ATCC CRL-2271) and CHLA255 (RRID: CVCL_AQ27) were cultured in Dulbecco’s Modified Eagle Medium (DMEM) or RPMI-1640 containing 10% foetal calf serum (FCS) and supplemented with 1% penicillin–streptomycin. The small interfering RNA (siRNA) of Sulf2 (Silencer Select SULF2, s31805, s31806) and its negative control (scramble, #AM4611) were obtained from Life Technologies. Transfection of siRNA was in the presence of lipofectamine RNAi Max (Life Technologies) according to the manufacturer’s instructions. The cells were cultured at 37 ˚C with 5% CO_2_ for 4 h and then the medium was changed to continue culture for 48 h. Western blot (WB) was used to detect the transfection efficiency with a mouse MAb against human SULF2 (2B4; Novus Biologicals #NBP1-36727, 1:1000 dilution). Membranes were analysed with the Li-Cor Odyssey infrared imager using an anti-mouse IgG IRdye 680 (Li-Cor #926-32220) as secondary antibody (1:5000).

### Cell migration assays

For the transwell migration assay, 1 × 10^4^ CHLA-255 and SK-N-BE(2) cells transfected with siRNA Sulf2 (Silencer Select SULF2, s31805, s31806; negative control AM4611, from Life Technologies) as previously described [15] were plated in 24-well, 8.0 μm pore size (Becton Dickinson, NJ, USA; #353182), and exposed to cell culture medium containing 20% FBS added to the lower chamber of the transwell dish. After 12 h migration, transwell inserts were removed and non-migratory cells at the upper side of the filter were swabbed off. Transwell filters were then fixed in 4% paraformaldehyde (PFA) solution in PBS and stained with 1% toluidine blue. Filters were cut free and mounted on slides for microscopic examination.

### Immunostaining

NBL cell lines were grown on a glass coverslip, fixed with 4% PFA and incubated overnight at 4 °C with HS phage display antibody 3A8V (a gift from Professor A. van Kuppevelt, Nijmegen, The Netherlands) diluted 1:5 in 1% goat serum in PBS. Bound antibody was detected with rabbit polyclonal VSV-G tag antibody (Invitrgen, Cat # PA1-30138), diluted 1:200 in 1% (v/v) goat serum. Secondary antibodies were anti-rabbit IgG antibodies conjugated to AlexaFluor-488 diluted 1:500. Hoechst 33,342 was used as a nuclear counterstain. Controls were the omission of the primary antibody. Images were captured using an LSM 719 confocal microscope with a 63x/1.4 Plan-APOCHROMAT oil immersion objective lens (Carl Zeiss Microscopy). To examine the expression of HS we used the mouse monoclonal anti-HS antibody 10E4 (Seikagaku, Tokyo, Japan), which recognises an epitope associated with N-sulphated domains of native HS [19, 20]. The HS antibody 10E4 was used at a 1:100 (v/v) dilution and after an overnight incubation at 4 °C, an anti-mouse AlexaFluor 488 IgG secondary antibody was used. In control sections to confirm the 10E4 antibody specificity, endogenous HS was removed by heparinase I (EC 4.2.2.7, Ibex technologies, Cat# 50 − 010), heparinase II (unassigned EC, Cat# 50 − 011) and heparinase-III (EC 4.2.2.8, Cat# 50 − 012) digestion (2.5 mU) for 1 h at 37 °C. Formalin-fixed paraffin-embedded sections of 65 primary human NBL tumours were obtained from the Children’s Oncology Group (COG) Neuroblastoma Biology Study with the approval of the Children’s Hospital Los Angeles (CHLA) Institutional Review Board without patient identification [15]. Tissue Sections (5 μm thickness) underwent heat antigen retrieval using 0.1 mM sodium citrate buffer, pH 6, and were incubated overnight at 4 °C with the 10E4 antibody at a 1/100 dilution. Conjugated anti-mouse IgG antibody and avidin/biotin complex (Vectastain ABC kit, Vector Laboratories) and 3,4-diaminobenzidine (DAB brown) was used to visualize antibody binding. Mayer’s haematoxylin was used to counterstain cell nuclei. To confirm antibody epitope binding, control sections were heparinase I-III digested. Immunohistochemical quantification of 10E4 staining was performed by colour deconvolution, as previously described [15]. Briefly, digital images of 3,3′-diaminobenzidine (DAB)-stained sections were analysed using Fiji/ImageJ. Brown DAB staining was separated from blue haematoxylin counterstaining using the H DAB colour deconvolution matrix, and the resulting DAB channel was used to quantify staining by optical density (OD).

### Statistical analysis

Differences between groups were compared using Student’s *t*-test or ANOVA as appropriate, with log10 transformation to normalise the data when necessary. Differences were considered statistically significant at *p* < 0.05. Statistical analyses were performed using GraphPad Prism (version 6.05).

## Results

### HS expression is abundant in MYCN-amplified NBL cells and is reduced following SULF2 knockdown

**Fig. 1:**
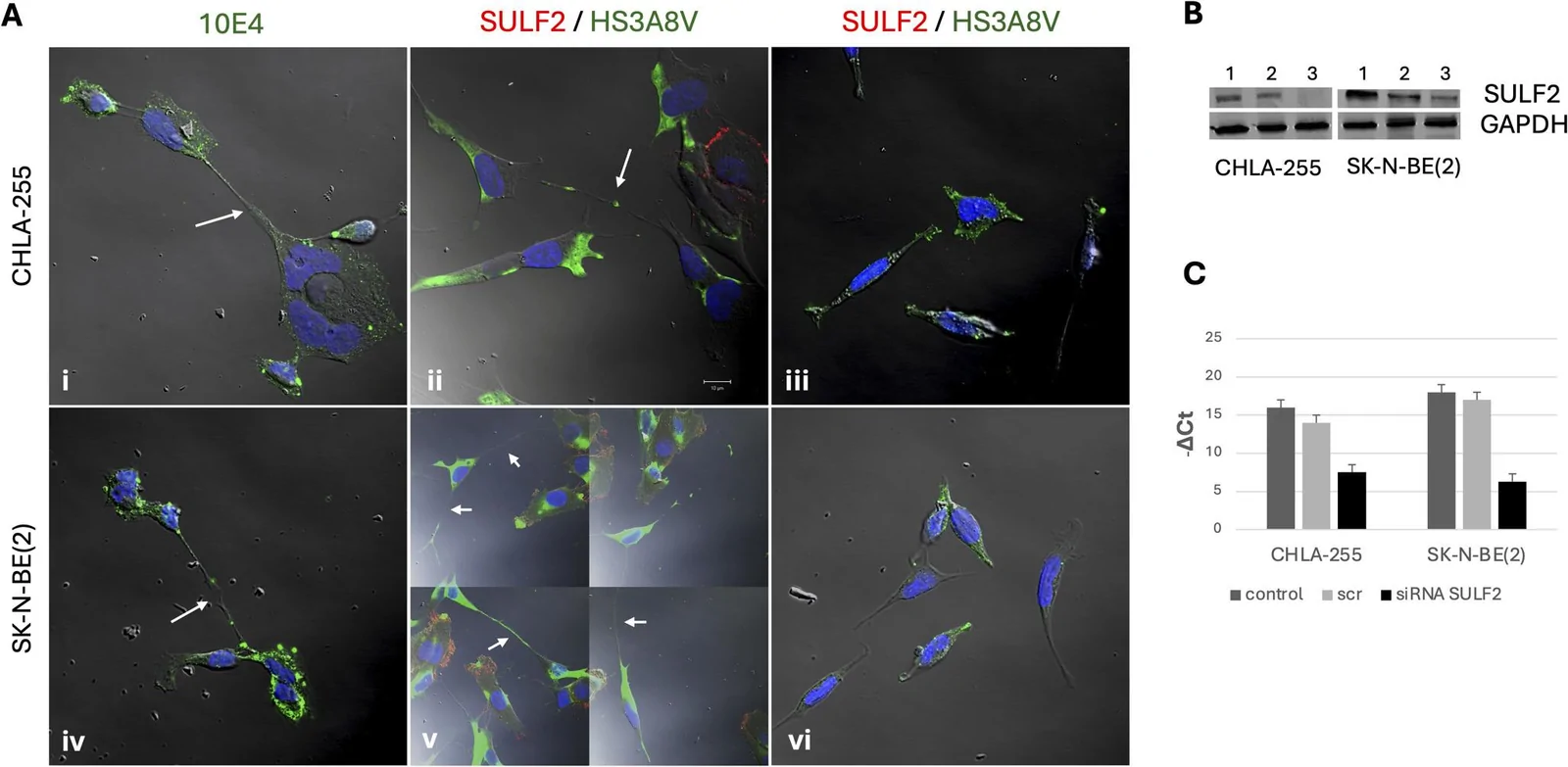
HS expression and SULF2 knockdown in NBL cells. **A** representative immunofluorescence images of MYCN-NA CHLA-255 and MYCN-A SKNBE2 cells. Panels i and iv show staining with the anti-HS antibody 10E4 (green). Panels ii and v show staining with the anti-HS phage-display antibody HS3A8V (green) and SULF2 (red); white arrows indicate long HS-positive intercellular extensions. Panels iii and vi show HS3A8V staining following siRNA-mediated SULF2 knockdown. Nuclei were counterstained with Hoechst 33,342 (blue). **B** Efficiency of SULF2 knockdown in CHLA-255 and SK-N-BE(2) cells assessed by Western blotting, lanes 1–3 represent untreated control, scrambled siRNA control and SULF2 siRNA, respectively; and **C** by qRT-PCR **C** qRT-PCR data are presented as means − ΔCt ± SEM from three aliquots per sample from three independent experiments, with GAPDH used as the reference gene. Higher − ΔCt values indicate higher levels of SULF2 expression

The distribution of HS was investigated in the MYCN-non-amplified (MYCN-NA) CHLA-255 and MYCN-amplified (MYCN-A) A SK-N-BE(2) NBL cell lines using the 10E4 antibody, which recognises native HS epitopes. 10E4 staining demonstrated abundant cell-surface HS in both cell lines, with more prominent staining in SK-N-BE(2) cells compared with CHLA-255 cells (Fig. 1A, i and iv). HS staining with 10E4 was also observed along intercellular extensions connecting spatially separated NBL cells (*white arrows*), (Fig. 1A, i and iv). HS distribution was further examined using the phage display antibody HS3A8V. HS3A8V staining was more prominent in MYCN-A SK-N-BE(2) cells than in MYCN-NA CHLA-225 cells and was particularly evident along intercellular extensions (Fig. 1A, ii and v, *white arrows*). Following siRNA mediated SULF2 knockdown, these long intercellular extensions were markedly reduced or no longer apparent, and were accompanied by an apparent reduction in cell-surface HS staining (Fig. 1A, iii and vi). The efficiency of SULF2 knockdown was confirmed in both NBL cell lines at the protein (Fig. 1B) and mRNA level (Fig. 1C), with a particularly marked effect in MYCN-A SK-N-BE(2) cells. The transfection efficiency was also noticed in the marked reduction to absence of SULF2 staining (Fig. 1A, iii and vi). Additionally, the two HS antibodies displayed distinct staining patterns with the 10E4 epitope showing predominantly punctate cell-surface localisation (Fig. 1A and iv), whereas 3A8V immunoreactivity was more continuously distributed along the cell membrane and intercellular extensions (Fig. 1A ii and v). These differences are consistent with the recognition of structurally distinct HS domains and may reflect differences in epitope distribution and accessibility rather than differences in overall HS abundance.

### SULF2 knockdown reduces NBL cell migration, with a greater effect in MYCN-amplified cells

**Fig. 2:**
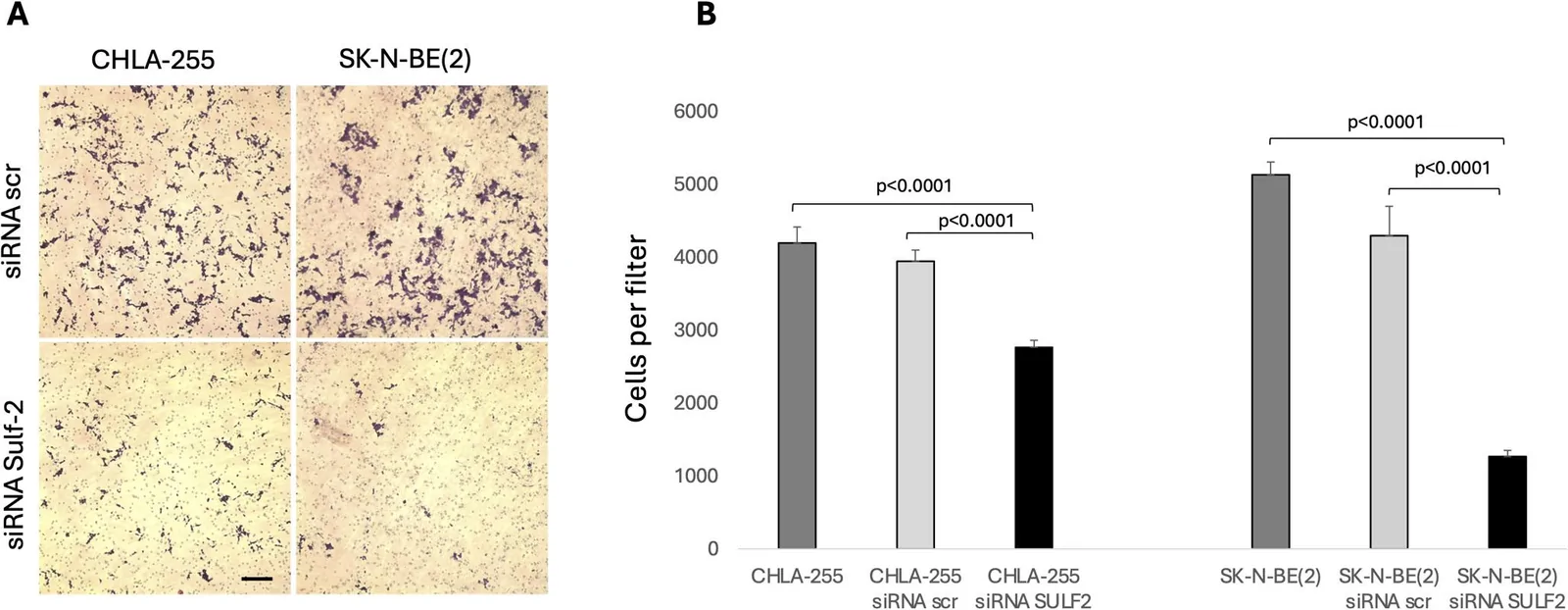
SULF2 knockdown reduces neuroblastoma cell migration. **A** Representative light micrographs of Transwell filters showing migration of CHLA-255 and SK-N-BE(2) cells following transfection with scrambled control (siRNA scr) or SULF2 siRNA and incubation for 12 h. Scale bar, 100 μm. **B** Quantification of migrated cells per filter demonstrating reduced migration following SULF2 knockdown, with a more pronounced reduction in MYCN-A SK-N-BE(2) cells. Data are presented as means ± SD. Statistical significance is indicated on the graph

SULF2 knockdown by siRNA significantly reduced in-vitro NBL cell migration in both cell lines (Fig. 2A and B). In the MYCN-NA CHLA-255 cells, migration decreased from 3797 ± 155 to 2768 ± 95 cells/filter (mean difference 1029 cells/filter, 95% CI 829–1228; *p* < 0.0001). In MYCN-A SK-N-BE(2) cells, migration decreased from 4298 ± 403 to 1268 ± 85 cells/filter (mean difference 3030 cells/filter, 95% CI 2584–3477; *p* < 0.0001). Following SULF2 knockdown, migration was significantly lower in SK-N-BE(2) cells than in CHLA-255 cells (mean difference 1500 cells/filter, 95% CI 1380–1620; *p* < 0.0001), (Fig. 2B).

###  10E4 reactive HS is increased in MYCN-amplified primary human NBL tumours

**Fig. 3:**
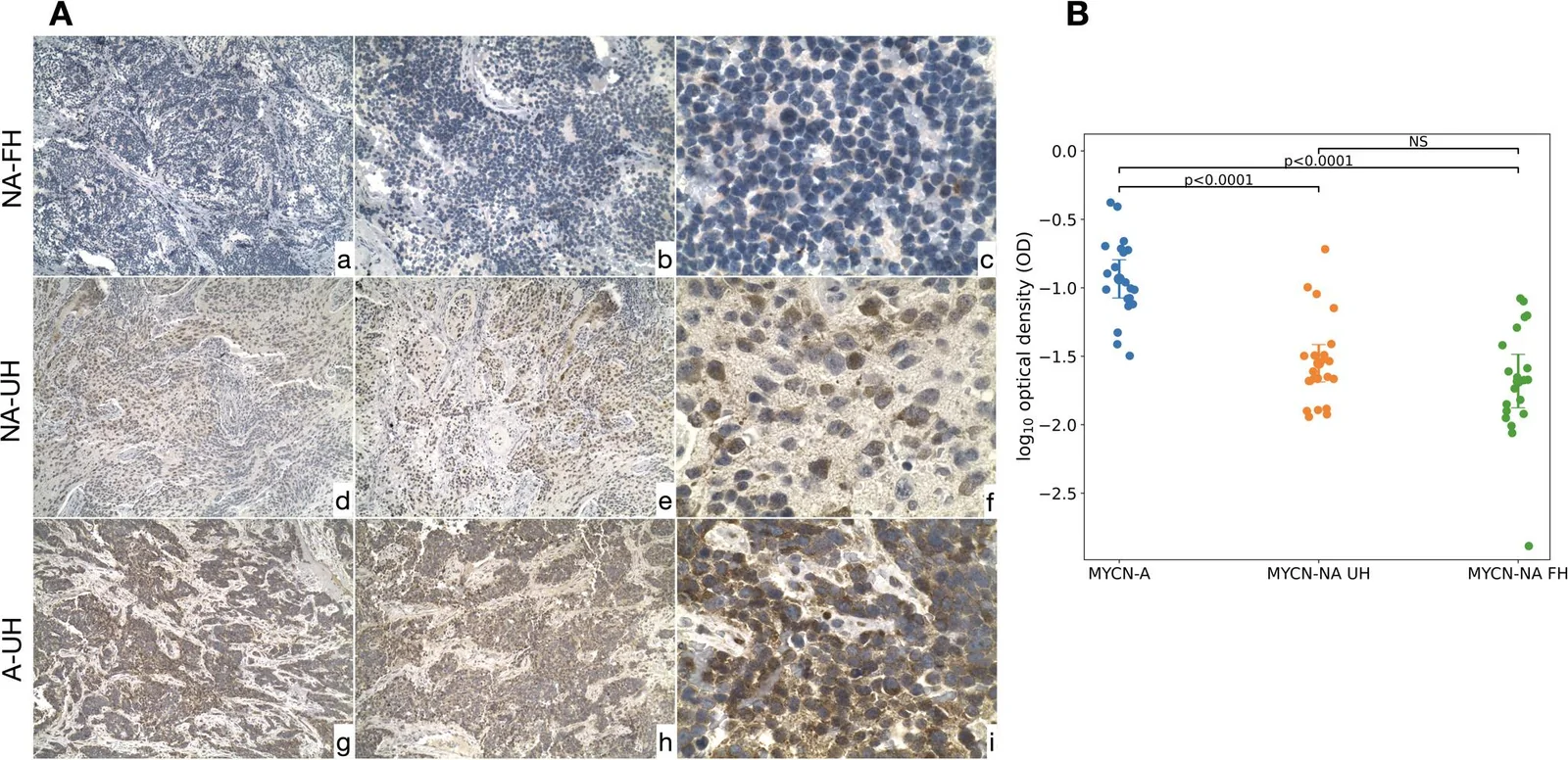
Increased HS expression in MYCN-amplified primary human NBL primary tumours. **A** Representative sections of primary human NBL tumours immunostaining with anti-HS antibody 10E4. (a-c), MYCN-NA tumours with FH, (d-f) MYCN-NA tumours with UH, and (g-i) MYCN-A tumours with UH. Scale bar, 20 μm. **B** Quantification of 10E4 immunoreactivity by colour deconvolution analysis. Optical density (OD) was calculated from 10 fields (20x magnification) per tumour section and log_10_-transformed for statistical analysis. Individual points represent individual tumours; data are presented as means ± SD

To determine whether the HS expression patterns observed in established NBL cell lines are also present in human tumours, 65 primary human NBL tissue specimens were analysed histologically and by immunohistochemistry using the anti HS antibody 10E4. The cohort comprised 22 MYCN-A tumours with unfavourable histology (A-UH), 23 MYCN-NA tumours with UH (NA-UH), and 20 MYCN-NA with favourable histology (NA-FH), (Fig. 3A). 10E4 immunoreactivity was predominantly localised to the tumour cell surface and surrounding extracellular matrix and was particularly prominent in high-risk MYCN-A-UH tumours (Fig. 3, g-i). MYCN-NA tumours with a FH had very weak 10E4 immunostaining (Fig. 3, a, b).

Quantitative colour deconvolution analysis demonstrated significantly higher 10E4 immunoreactivity in MYCN-A NBL tumours than in either MYCN-NA-UH or MYCN-NA-FH tumours (one-way ANOVA of log₁₀-transformed optical-density [OD] values, *p* < 0.0001), (Fig. 3B). Expression was 4.13-fold higher in MYCN-A than in MYCN-NA tumours with UH (95% CI 2.30–7.42; *p* < 0.0001) and 5.58-fold higher than in MYCN-NA tumours with FH (95% CI 3.04–10.23; *p* < 0.0001). No significant difference was observed between the MYCN-NA tumours with UH and FH groups (1.35-fold difference, 95% CI 0.75–2.43; *p* = 0.439).

## Discussion

The present study demonstrates that HS expression and distribution are associated with the aggressive MYCN-amplified phenotype of NBL and supports a functional relationship between HS remodelling and SULF2 activity. HS epitopes recognised by 10E4 and HS3A8V were abundant on the surface of NBL cells and particularly prominent in MYCN-A SK-N-BE(2) cells. The distinct spatial distribution of 10E4-and HS3A8V reactive epitopes further highlight the structural heterogeneity of cell surface HS. The predominantly punctate distribution of 10E4, compared with the more continuous HS3A8V staining along cellular extensions, may reflect differences in the organisations, sulphation and accessibility of individual HS domains. As HS binding antibodies recognise structurally distinct epitopes whose availability may also be influenced by protein binding, these staining patterns should not be interpreted simply as differences in total HS abundance. SULF2 knockdown was associated with altered HS staining, loss of prominent HS-positive intercellular extensions and significantly reduced cell migration, with the greatest effect in MYCN-A cells. Importantly, analysis of 65 primary human NBL tumours demonstrated substantially higher 10E4 immunoreactivity in MYCN-A than in MYCN-NA tumours. Together, these findings suggest that MYCN-A NBL is characterised by a particular HS phenotype, involving variable degrees of 6-O-sulphation within structurally heterogeneous HS chains, and that SULF2-mediated HS remodelling (resulting in reduced and/or altered patterns of 6-O-sulphation) may contribute to aggressive tumour behaviour.

Heparan sulfate is an important component of the tumour microenvironment, where its structural heterogeneity enables interactions with growth factors, cytokines and extracellular matrix proteins [8–10]. The biological consequences of these interactions depend on HS structure and cellular localisation. This may be particularly relevant in NBL, where interactions between neuroblasts and their microenvironment influence tumour behaviour [5–7]. Knelson et al. demonstrated enrichment of several HSPGs within the Schwannian stroma and showed that soluble HSPGs promoted neuroblast differentiation, suggesting a tumour-suppressive function of stromal HS [12]. Our findings are not necessarily contradictory; rather, they suggest that the biological effects of HS may depend on its cellular source, spatial distribution and structural characteristics. Whereas stromal HS may promote differentiation, distinct tumour-associated HS structures may support malignant behaviour. The complementary staining patterns with 10E4 and HS3A8V supports this concept. 10E4 recognises native HS domains containing mixed N-acetylated and N-sulphated sequences [19, 20], whereas the phage-display antibody HS3A8V recognises structurally distinct HS epitopes with binding characteristics dependent on saccharide sequence and higher-order conformation [16–18]. Thus, differences in staining with these antibodies may reflect differences in HS structure and epitope accessibility rather than simply differences in the amount of HS present.

These findings are consistent with our previous demonstration of a close relationship between SULF2 and MYCN in NBL [15]. SULF2 modifies extracellular ligand–HS interactions by selectively removing 6-O-sulphate groups from HS [13]. We previously demonstrated that SULF2 expression was associated with MYCN amplification and adverse outcome, and that modulation of SULF2 affected NBL cell survival [15]. The present study extends these observations by linking SULF2 to HS distribution and cell migration. The particularly marked reduction in migration following SULF2 knockdown in MYCN-A cells suggests that SULF2-dependent HS remodelling may contribute to a more motile tumour phenotype. A further observation was the localisation of HS to long intercellular extensions, particularly in MYCN-A cells. These structures were markedly reduced following SULF2 knockdown. Although their morphology suggests a potential role in tumour cell interactions, their precise nature and function remain undetermined, and the present findings do not demonstrate direct intercellular communication. Further structural and functional studies are required to determine whether HS remodelling contributes to their formation or maintenance. The altered staining following SULF2 knockdown may therefore reflect not only changes in HS abundance but also changes in the structural organisation or accessibility of specific HS epitopes.

The findings in primary human tumours strengthen the relevance of the cell-line observations. 10E4 immunoreactivity was over 4-fold higher in MYCN-A tumours than in MYCN-NA tumours with unfavourable histology and over 5-fold higher than in those with favourable histology, whereas the two MYCN-NA groups did not differ significantly. Thus, within this cohort, increased 10E4 immunoreactivity was more closely associated with MYCN amplification than with histological classification. Together with the cell line findings and our previous observation linking MYCN and SULF2 [15], these results support a potential MYCN–SULF2–HS relationship in aggressive NBL. However, these associations do not establish a direct causal relationship between MYCN, SULF2 mediated HS remodelling and tumour behaviour.

Several limitations should be acknowledged. Only two NBL cell lines were examined, limiting the extent to which the findings can be generalised across the molecular heterogeneity of NBL. The immunofluorescence observations were predominantly qualitative. Changes in antibody staining cannot distinguish altered HS abundance from changes in epitope structure or accessibility, and the intercellular extensions were not functionally characterised. Furthermore, the primary tumour cohort did not include MYCN-A tumours with favourable histology.

In conclusion, MYCN-A NBL cells and primary tumours demonstrate a distinct HS phenotype associated with SULF2 activity and cell migration. Together with previous evidence linking SULF2 to MYCN and NBL cell survival, these findings suggest that SULF2-mediated remodelling of HS 6-O-sulphation may contribute to aggressive NBL biology. Finally, as an extracellular enzyme, SULF2 represents a potentially accessible therapeutic target, and selective inhibition of SULF2-mediated HS remodelling warrants further investigation in high-risk NBL [21].

## Data Availability

No datasets were generated or analysed during the current study.
